# Prostatic urethral lift (UroLift): a real-world analysis of outcomes using hospital episodes statistics

**DOI:** 10.1186/s12894-021-00824-5

**Published:** 2021-04-07

**Authors:** Toby Page, Rajan Veeratterapillay, Kim Keltie, Julie Burn, Andrew Sims

**Affiliations:** 1 The Newcastle Upon Tyne Hospitals NHS Foundation Trust, Newcastle upon Tyne, UK; 2 Faculty of Medical Sciences, Translational and Clinical Research Institute, University of Newcastle Upon Tyne, Newcastle upon Tyne, UK

**Keywords:** Prostatic urethral lift, BPH, LUTS, Urinary retention, UroLift

## Abstract

**Background:**

To determine real-world outcomes of prostatic urethral lift (UroLift) procedures conducted in hospitals across England.

**Methods:**

A retrospective observational cohort was identified from Hospital Episode Statistics data including men undergoing UroLift in hospitals in England between 2017 and 2020. Procedure uptake, patient demographics, inpatient complications, 30-day accident and emergency re-attendance rate, requirement for further treatment and catheterization were captured. Kaplan–Meier and hazard analysis were used to analyse time to re-treatment.

**Results:**

2942 index UroLift procedures from 80 hospital trusts were analysed; 85.3% conducted as day-case surgery (admitted to hospital for a planned surgical procedure and returning home on the same day). In-hospital complication rate was 3.4%. 93% of men were catheter-free at 30 days. The acute accident and emergency attendance rate within 30 days was 12.0%. Results of Kaplan Meier analysis for subsequent re-treatment (including additional UroLift and endoscopic intervention) at 1 and 2 years were 5.2% [95% CI 4.2 to 6.1] and 11.9% [10.1 to 13.6] respectively.

**Conclusions:**

This real-world analysis of UroLift shows that it can be delivered safely in a day-case setting with minimal morbidity. However, hospital resource usage for catheterization and emergency hospital attendance in the first 30 days was substantial, and 12% required re-treatment at 2 years.

**Supplementary Information:**

The online version contains supplementary material available at 10.1186/s12894-021-00824-5.

## Background

Prostatic urethral lift (PUL) is a minimally invasive treatment for men with lower urinary tract symptoms (LUTS), which involves placing non-absorbable sutures with a nitinol prostate capsular anchor and a stainless steel urethral end piece to mechanically open the anterior prostatic fossa and disobstruct the urethra [1]. As the treatment does not use thermal energy to excise or ablate tissue it also reduces some adverse effects, such as erectile dysfunction and ejaculatory dysfunction, which can be associated with traditional treatments such as TURP [2]. Other minimally invasive treatments are available; prostate artery embolization (PAE) and iTIND also avoid the use of thermal energy, Rezum therapy uses steam heat energy to remove prostate tissue, whilst Aquablation uses high pressure water jet to remove adenoma. Currently in the UK there is no consensus as to which treatment should be offered to which men and at what point in the treatment pathway. Recent UK National audits suggest the majority of men are still treated with traditional TURP, and that less than 10% of men are being treated with PUL, even less with PAE and Rezum, and that Aquablation is only offered at one centre in the UK [3].

PUL marketed as UroLift (manufactured by NeoTract Inc.) was first performed in the United Kingdom (UK) as part of the commercially sponsored BPH-6 trial [4]. The UK National Institute for Health and Care Excellence (NICE) published interventional procedures guidance for urethral lift in January 2014, recommending its use for the treatment of men with lower urinary tract symptoms [5], and Medical Technology Guidance on the UroLift System in September 2015 [6]. This recommended UroLift as an alternative to surgical procedures, in a day-case surgery setting, in men over the age of 50, who have a prostate of less than 100 ml without an obstructing middle lobe. Further studies support the wider use of PUL in men with obstructive median lobes [7], large prostates [8] and in men with retention [9]. Yet despite the positive published outcomes of UroLift, adoption of the procedure in the NHS has been slow. To encourage widespread adoption, UroLift was added to the Innovation Technology Tariff (ITT) in April 2017, and subsequently selected as a Rapid Uptake Product by the National Health Service (NHS) Accelerated Access Collaborative in 2018 [10]. Both schemes aim to support adoption of innovative technologies within the NHS.

Hospital Episode Statistics (HES) is a data warehouse containing episodes of care under a single consultant for patients at NHS hospitals in England. HES datasets include Admitted Patient Care (all admissions including day-case procedures), outpatient appointments and attendance at accident and emergency departments [11]. Clinical coding of procedures uses the Classification of Intervention and Procedures, (OPCS-4) and coding of diagnoses uses the International Classification of Diseases (ICD-10). A specific procedure code was introduced into the UK National Clinical Coding Standards for UroLift in 2017 (“M68.3: Endoscopic insertion of prosthesis to compress lobe of prostate”) enabling robust identification of procedures in HES data from that point.

The aim of this study is to use national administrative data from HES to determine uptake as well as real-world in-hospital and longitudinal outcomes of prostatic urethral lift (UroLift) procedures conducted in an NHS hospital setting in England.

## Methods

Episodes of UroLift implantation were identified from the presence of procedure code “M68.3: Endoscopic insertion of prosthesis to compress lobe of prostate” in the HES Admitted Patient Care (APC) dataset with a discharge date between 1st April 2017 and 31st January 2020. This dataset also includes day-case surgeries where the patient is admitted to hospital for a planned surgical procedure and returns home on the same day. Individual episodes of care from HES were aggregated into admissions (single periods of care within a treating hospital) [12]. Analysis was restricted to the earliest UroLift implantation admission for each patient (index admission) conducted within NHS hospitals, which also included a diagnosis code relating to benign prostate hyperplasia (Additional file 1). Those discharged after 1st January 2020 were excluded to ensure 30 day follow up.

Pseudonymised data from HES and the Civil Registration (formerly, the Office of National Statistics) Mortality datasets were supplied under Data Access Request Service (DARS) agreement DARS-NIC-170211-Z1B4J. No patient identifiable information was used and ethical approval was not sought or required. All scripts for applying eligibility criteria, data cleaning, processing and statistical analysis were written in the statistical programming language R [13].

Patient characteristics from the index UroLift procedural admission were summarised using descriptive statistics. Patients catheterized on admission were identified by the presence of ICD10 code Z96.0 “Presence of urogenital implants”. In-hospital outcomes included complications [14], catheterization due to retention (procedure code M47 “Urethral catheterization of bladder”), subsequent removal of the catheter (M47.3 “removal of catheter from bladder”), length of hospital stay and death.

All hospital activity (including APC episodes, day-case surgeries, outpatients, and accident and emergency attendances) and all-cause mortality occurring after discharge from the index UroLift implantation were extracted for the cohort. Outcomes at 30-days included catheter status by analyzing catheterization code in both APC and outpatient attendances. Longitudinal outcomes included retreatment (Additional file 2), other bladder/prostate intervention (Additional file 3) and all-cause mortality. Kaplan–Meier analysis was applied to the time from the discharge date of the index UroLift procedure to the date of retreatment (by UroLift of other endoscopic interventions) or date of death. The timing of events was estimated using the hazard function [15]. Patients with no events and known to be alive at the end of the study were considered censored.

## Results

### Cohort identification

**Fig. 1:**
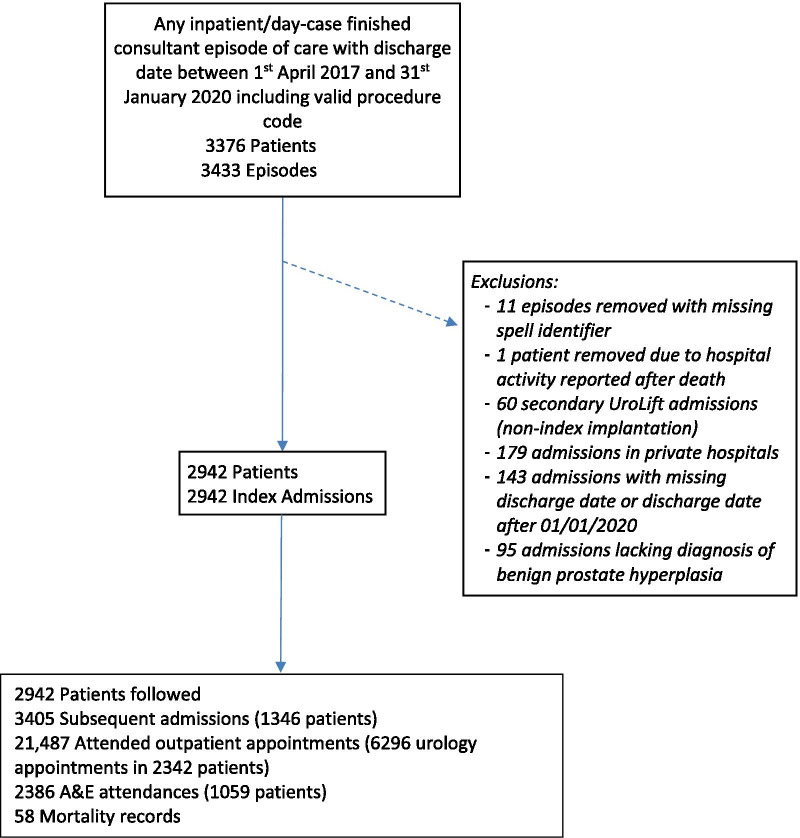
HES identification of study population

A total of 3433 hospital episodes of care from 3376 patients were identified by the initial search in HES APC. Index UroLift admissions were identified for 3359 patients; exclusions included: 179 treated within private centres, 143 missing discharge date or discharged after 1^st^ January 2020, 95 without eligible diagnoses of BPH (Fig. 1). Following exclusions, 2942 UroLift procedures from 2942 patients, treated across 80 NHS hospital trusts remained for analysis; with an increase in procedure uptake during the study period (Additional file 4).

### Patient demographics

The median age at implantation was 69 years [Q1:Q3, 61 to 75] with 108 patients (3.7%) aged less than 50 years at the time of their index UroLift implantation, Table 1. The majority of patients (n = 2509, 85.3%) had the procedure as a day-case surgery.

**Table 1:** Demographics and in-hospital outcomes

	Patient demographics (n = 2942)
Demographics	
Age, years median, (Q1:Q3); [min–max]	69 (61:75) [35 to 97]
Elective admission (%)	2938 (99.9%)
Day case admission (%)	2317 (78.8%)
Comorbidities (ICD-10)^a^	
Diabetes (E10–E14)	421 (14.3%)
Hypertensive disease (I10–I15)	1066 (36.2%)
Ischaemic heart disease (I20–I25)	373 (12.7%)
Heart failure (I50)	52 (1.8%)
Other chronic obstructive pulmonary disease (J44)	168 (5.7%)
Catheter in place at admission	82 (2.8%)
Outcomes	
In-hospital complication (%)	99 (3.4%)
Catheterisation due to retention (%)	135 (4.6%)
Length of stay	
Day case	2509 (85.3%)
1 night	354 (12.0%)
More than 1 night	79 (2.7%)
In-hospital deaths (%)	0 (0.0%)

^a^ICD10 codes in any DIAG01-20 position, not exclusive

### In-hospital (procedural) outcomes

Inpatient complications were recorded for 99 patients (n = 3.4%) with the most commonly reported complication being urinary retention (n = 40, 1.4%), followed by haematuria/haemorrhage (n = 26, 0.9%) (Additional file 5). Inpatient infective complications were coded in 3 cases (0.1%) and there was one coded case of bladder injury. No in-hospital deaths occurring during the procedural admission were identified.

Eighty-two patients (2.8%) already had a catheter in place for retention at admission and 135 (4.6%) additional patients were catheterized during their admission. In our cohort of 2942 men, 2747 (93.4%) were catheter-free at discharge.

### Longitudinal (post-discharge) outcomes

Within 30 days of discharge from the UroLift implantation, 394 patients (13.4%) had 496 hospital admissions (reasons for readmission described in Additional file 6); 250 of which were an emergency (50%). A total of 881 patients (29.9%) attended 1452 outpatient appointments within 30 days. Of these, 336 patients attended 435 urology outpatient appointments within 30 days (106 (24.3%) appointments for removal of catheter, Additional file 7). A total of 352 patients (12.0%) attended A&E 472 times within 30 days (168 A&E attendances in 152 patients resulted in admission). Two patients died within 30 days of UroLift implantation—with main cause of death including “Infection following a procedure” (contributory factors including sepsis) and “Atrial fibrillation and atrial flutter, unspecified” (contributory factors including congestive heart failure, COPD and type 2 diabetes). A total of 2737 men (93.0%) were catheter-free at 30-days.

Analysis of longitudinal outcomes was conducted for all 2942 patients discharged post-UroLift implantation, with a total follow-up of 1,313,627 patient days. The median follow-up per patient was 424 days (Q1:Q3 of 211:653, range 11–1032 days). Throughout follow-up, a total of 3405 subsequent all-cause admissions from 1346 patients were recorded. In addition, 243/2737 men who were catheter-free at 30 days required subsequent catheterization (8.9%) and 42/205 men who had a catheter in place at 30 days had subsequent catheterization (20.5%).

**Fig. 2:**
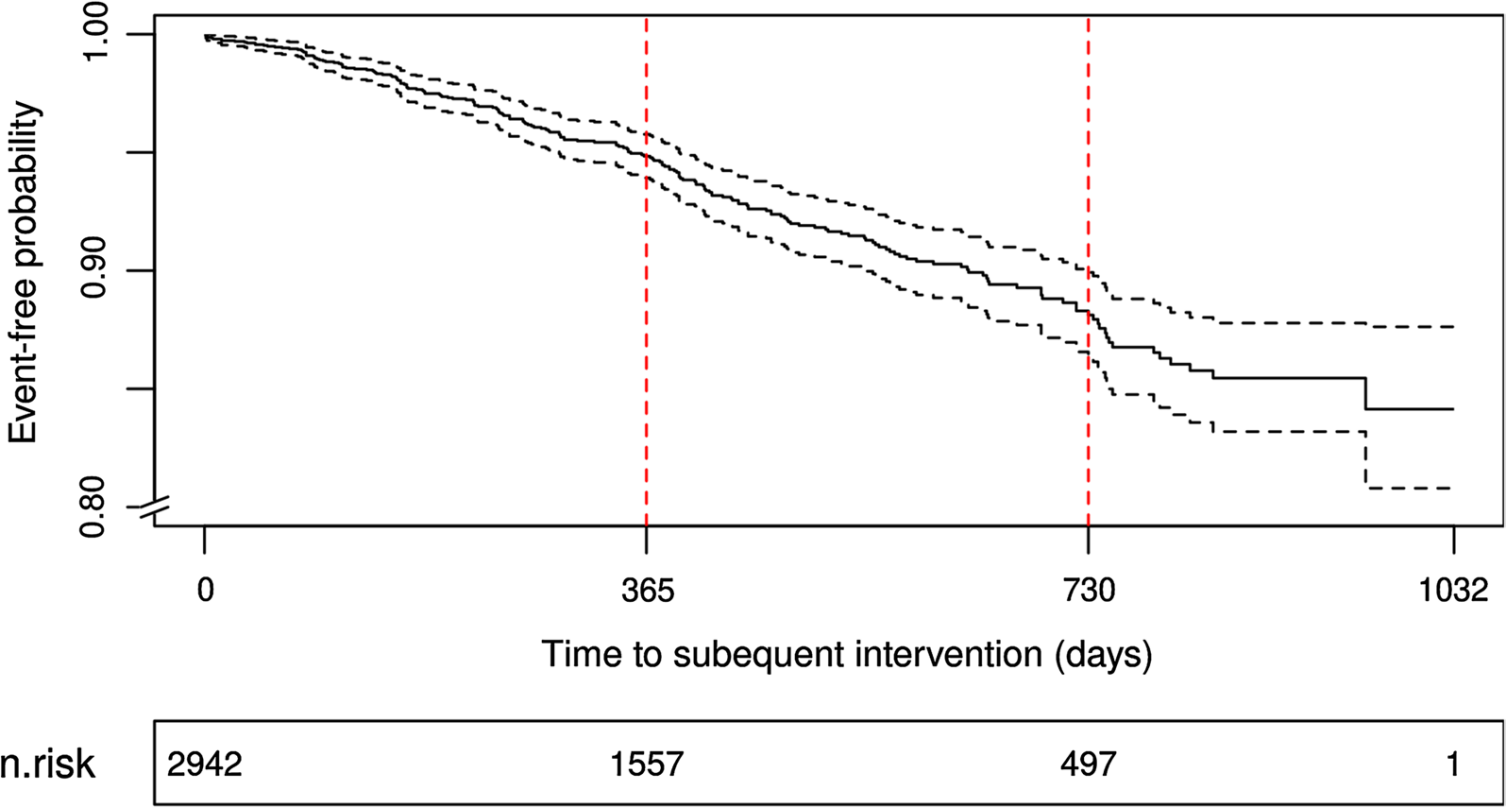
Kaplan Meier plots showing re-intervention rates

During follow-up, 206 patients required retreatment with 57 patients requiring further UroLift intervention and 158 patients requiring endoscopic intervention (Table 2; Additional file 8). Additional interventions described in Additional file 3. Subsequent UroLift treatment at 1 and 2 years was 1.5 [95% CI 1.0 to 2.0]% and 3.0 [2.1 to 3.8]% respectively, subsequent endoscopic treatment (excluding UroLift) was 3.9 [3.0 to 4.7]% and 9.5 [7.9 to 10.1]% (Table 2). Overall retreatment was 5.2% [95% CI 4.2 to 6.1] and 11.9% [10.1 to 13.6] at 1 and 2 years respectively (Fig. 2, Additional file 9). Two patients died within 30 days of UroLift implantation; the mortality rate in our cohort at year 1 and year 2 post-procedure were 1.4 [0.9 to 1.9]% and 3.1 [2.2 to 4.0]% respectively.

**Table 2:** Longitudinal outcomes at 1 and 2 years

	Total events	1 year event-free probability [95% CI]	2 year event-free probability [95% CI]
Retreatment	206	0.948 [0.939 to 0.958] (n = 1557)	0.881 [0.864 to 0.899] (n = 497)
UroLift	57	0.985 [0.980 to 0.990] (n = 1620)	0.970 [0.962 to 0.979] (n = 543)
Endoscopic	158	0.961 [0.953 to 0.970] (n = 1579)	0.905 [0.889 to 0.921] (n = 508)
All-cause mortality	58	0.986 [0.981 to 0.991] (n = 1647)	0.969 [0.960 to 0.978] (n = 557)

## Discussion

This HES analysis represents the largest cohort study of UroLift in a NHS hospital setting, with robust identification of procedures, comprehensive coverage across England, median follow-up of 1.2 years [min 11 days; max 2.8 years]. Our study demonstrates an increase in uptake of the procedure across the NHS during the study period, with 80 NHS trusts providing the procedure in England. In our study 3.7% of patients were aged less than 50 years old, this is higher than the rate reported in a retrospective case review conducted in the US and Australia (17 in 1413, 1.2%) [16]. However, given the major benefit of PUL is to maintain sexual function there continues to be a strong argument for treating younger men who may wish to preserve normal ejaculation for conception. Our results suggest a potential selection bias towards older men as a new procedure is introduced into practice, which may represent a learning curve as clinicians become comfortable with the technique and its results. Additionally in the UK the majority of men opt for pharmaceutical treatment rather than procedure based treatment for mild to moderate symptoms due to national guidelines.

We confirmed that the majority of procedures were conducted as day-case surgeries (85.3%) and that there was a low rate of in-hospital complications (3.4%). This is in keeping with previous reports that UroLift has several advantages including the ability to be undertaken under local anesthesia as a day case, a short learning curve and shorter procedure time (compared with traditional bladder outlet procedures) [17]. The majority (> 95%) of complications reported in the literature have been Clavien grade 1 with the most common being pelvic pain and dysuria [17]. The complication rate we found was low but we are not able to directly compare ICD-10 classification with Clavien–Dindo without introducing reporter bias in the assessment of the severity of the ICD-10 code in the absence of detailed patient notes. The most common adverse event we noted was urinary retention followed by bleeding.

By day 30, 205 men (7.0%) remained catheter dependent, and an additional 243 men required catheterization after 30 days (variable follow-up). In the LIFT study, 32% of patients required catheterization for failed voiding trial with a mean catheter duration of 0.9 days average for the whole cohort of 206 patients across the trial [1]. In the BPH-6 study, 45% of UroLift patients had a postoperative catheter for more than 24 h [4]. The post-operative catheterization rate we report is lower than in the two trials and may represent more cautious patient selection for implementation of a new procedure in an NHS setting compared to a trial setting, however the clinical profile of patients cannot be determined from HES coding. The lower rate may also be due to increasing clinician confidence and experience in the post-operative period after treatment.

Although the overall reported complication rate from UroLift is low, there is a paucity of longitudinal data on emergency re-presentations in the real-life setting. We noted a large proportion of our cohort (12%) attended A&E within 30 days of their PUL procedure. This will be an overestimate of complications, as some attendances maybe unrelated to UroLift, nevertheless it represents an upper limit used to demonstrate hospital service usage following UroLift implantation. However, there is little published data for comparable bladder outlet procedures for A&E admissions in the UK.

Due to large cohort size, our study is able to provide a more robust estimate of retreatment rates (11.9% at 2 years) following UroLift implantation. Retreatment rates were higher than those found in patients randomized to UroLift (n = 140) in the LIFT study (7.5% [95% CI 3.8 to 13.6%] at 2 years [18], but similar to the outcomes at 3 years, 10.7% [95% CI 6.3 to 17.3%] [19]. This may represent a lower threshold to offer retreatment outside of clinical trials.

Whilst this is a retrospective cohort study, it reports real-world outcomes from all UroLift procedures conducted across NHS hospitals in England, with comprehensive follow-up, which reduces both selection and reporting bias [11]. This approach allows normalization of outcome as it reflects both the high volume experienced surgeon and those surgeons who perform fewer cases. Given the large number of cases we have captured and described, the effect of outliers, surgical selection and retreatment bias is minimized, and the overall results offer a valid real-world reflection of the outcomes and hospital resource usage associated with PUL when conducted in an English NHS hospital.

There are however some limitations of using the HES data as it can only be used to produce overall performance indicators (e.g. readmission, complications and length of stay) and it does not allow assessment of all individual patient characteristics as some are not coded (e.g. severity of symptoms, size of prostate, presence of median lobe, International Prostate Symptom Score (IPSS), uroflowmetry, medication etc.) which makes it difficult for our study to comment on efficacy of UroLift. Additionally, due to lack of coding (both procedural and diagnosis) administrative data cannot be used to make meaningful comparisons of outcomes between the different prostate procedures (e.g. TURP, GreenLight, HoLEP etc.). However due to the creation of a procedure code introduced specifically for UroLift, and implemented in 2017, we have been able to use routine administrative data to identify and follow a cohort of men having this intervention across all NHS hospitals in England. This makes HES a powerful tool in investigating patient pathways, hospital resource usage and safety outcomes following an intervention due its comprehensive coverage of hospital activity.

## Conclusions

This HES data analysis of UroLift shows that it can be delivered safely in a day-case surgery setting with minimal morbidity. The hospital resource usage in terms of catheterization is lower than in published trial data but emergency hospital attendance in the first 30 days following UroLift implantation is higher than would have been expected for a minimally invasive treatment. The need for further surgical intervention seen in this study appears higher than reported in published trial data at 2 years which may have an impact on the health economic calculations used to assess the place of this treatment in NHS hospital pathways.

## Supplementary Information

**Additional file 1.** Online Resource 1: ICD codes for cohort identification.

**Additional file 2.** Online Resource 2: Subsequent interventions deemed as retreatment, identified by OPCS codes during follow-up.

**Additional file 3.** Online Resource 3: Additional interventions identified during follow-up (but not included within retreatment rates).

**Additional file 4.** Online Resource 4: Eligible UroLift procedure uptake in NHS hospitals in England between April 2017 and January 2020.

**Additional file 5.** Online Resource 5: In-hospital complications recorded during UroLift implantation. Note complications are not mutually exclusive as patients can have multiple complications.

**Additional file 6.** Online Resource 6: Reason for readmissions occurring within 30 days (using first diagnosis code).

**Additional file 7.** Online Resource 7: The main procedure conducted during urology outpatient appointment conducted within 30 days (using first procedure code).

**Additional file 8.** Online Resource 8: Retreatment procedures captured during follow-up.

**Additional file 9.** Online Resource 9: Hazard rate of retreatment (subsequent UroLift intervention or other endoscopic prostate intervention) following index UroLift implantation procedure. [Note: due to limited number of events kernel-density estimated smoothing was not appropriate, and piecewise exponential hazard function was applied].

## Data Availability

The data that support the findings of this study are available from NHS Digital but restrictions apply to the availability of these data, and so are not publicly available. Pseudonymised data from HES and the Civil Registration (formerly, the Office of National Statistics) Mortality datasets were supplied to the study team under Data Access Request Service (DARS) agreement DARS-NIC-170211-Z1B4J. Administrative Hospital Episodes Statistics (HES) data to reproduce results are available from the Health and Social Care Information Centre (HSCIC) via formal application process.

## Abbreviations

A&E: Accident and emergency
APC: Admitted patient care
HES: Hospital episode statistics
LUTS: Lower urinary tract symptoms
NHS: National Health Service
NICE: National Institute for Health and Care Excellence
PUL: Prostatic urethral lift
UK: United Kingdom
