# Supplementary material for: Prostatic urethral lift (UroLift): a real-world analysis of outcomes using hospital episodes statistics
Source: BMC Urol. 2021 Apr 7;21:55. doi: 10.1186/s12894-021-00824-5 (PMC8028737; doi:10.1186/s12894-021-00824-5)
Supplement: Supplementary file 1 — Additional file 1. Online Resource 1: ICD codes for cohort identification. [file 12894_2021_824_MOESM1_ESM.docx]

Online Resource 1: ICD codes for cohort identification

| ICD10 | Description |
| --- | --- |
| N40X | Hyperplasia of prostate |
| D29.1 | Benign neoplasm of male genital organs: Prostate |
| N32.0 | Bladder-neck obstruction |
| N32.8 | Other specified disorders of bladder |
| R33X | Retention of urine |
| R39.1 | Other difficulties with micturition |
| R39.8 | Other and unspecified symptoms and signs involving the urinary system |
