# Supplementary material for: Prostatic urethral lift (UroLift): a real-world analysis of outcomes using hospital episodes statistics
Source: BMC Urol. 2021 Apr 7;21:55. doi: 10.1186/s12894-021-00824-5 (PMC8028737; doi:10.1186/s12894-021-00824-5)
Supplement: Supplementary file 2 — Additional file 2. Online Resource 2: Subsequent interventions deemed as retreatment, identified by OPCS codes during follow-up. [file 12894_2021_824_MOESM2_ESM.docx]

Online Resource 2: Subsequent interventions deemed as retreatment, identified by OPCS codes during follow-up

|  | Description | OPCS coding |
| --- | --- | --- |
| *Subsequent UroLift intervention* | Urolift removal | M68.3 + [Y03.7\|Y15.7] |
|  | Urolift renewal | M68.3 + [Y03.2\|Y15.2] |
|  | Urolift adjustment | M68.3 + [Y03(1\|3\|4\|6\|8\|9)\|Y71(2\|3\|6\|7\|8\|9)\|Y15(1\|3\|4\|6\|8\|9) |
|  | Repeated Urolift | M68.3 |
| *Subsequent endoscopic intervention* | Endoscopic resection outlet of male bladder | M65.1 Endoscopic resection of prostate using electrotome  M65.2 Endoscopic resection of prostate using punch  M65.3 Endoscopic resection of prostate NEC  M65.4 Endoscopic resection of prostate using laser  M65.5 Endoscopic resection of prostate using vapotrode  M65.8 Other specified  M65.9 Unspecified |
|  | Other therapeutic endoscopic operations on outlet of male bladder | M66.1 Endoscopic sphincterotomy of external sphincter of male bladder  M66.2 Endoscopic incision of outlet of male bladder NEC  M66.3 Endoscopic injection of inert substance into outlet of male bladder  M66.8 Other specified other therapeutic endoscopic operations on outlet of male bladder  M66.9 Unspecified other therapeutic endoscopic operations on outlet of male bladder |
|  | Other therapeutic endoscopic operations on prostate | M70.4 Balloon dilation of prostate |
| *Subsequent open intervention* | Other open operations on outlet of male bladder | M64.1 Open resection of outlet of male bladder  M64.8 Other specified other open operations on outlet of male bladder  M64.9 Unspecified other open operations on outlet of male bladder |
|  | Open excision of prostate | M61.2 Retropubic prostatectomy  M61.3 Transvesical prostatectomy |
