# Supplementary material for: Prostatic urethral lift (UroLift): a real-world analysis of outcomes using hospital episodes statistics
Source: BMC Urol. 2021 Apr 7;21:55. doi: 10.1186/s12894-021-00824-5 (PMC8028737; doi:10.1186/s12894-021-00824-5)
Supplement: Supplementary file 3 — Additional file 3. Online Resource 3: Additional interventions identified during follow-up (but not included within retreatment rates). [file 12894_2021_824_MOESM3_ESM.docx]

Online Resource 3: Additional interventions identified during follow-up (but not included within retreatment rates).

| Procedure type | OPCS code and description | Number of patients | Number of procedures |
| --- | --- | --- | --- |
| Other therapeutic operations on prostate | M67.1 Endoscopic cryotherapy to lesion of prostate  M67.2 Endoscopic destruction of lesion of prostate NEC  M67.3 Endoscopic drainage of prostate  M67.4 Endoscopic removal of calculus from prostate  M67.5 Endoscopic microwave destruction of lesion of prostate  M67.6 Endoscopic radiofrequency ablation of lesion of prostate  M67.8 Other specified other therapeutic endoscopic operations on prostate  M67.9 Unspecified other therapeutic endoscopic operations on prostate | 0  3  0  0  0  0  6  0 | 0  3  0  0  0  0  6  0 |
| Other open operations on outlet of male bladder | M64.2 Implantation of artificial urinary sphincter into outlet of male bladder  M64.3 Insertion of prosthetic collar around outlet of male bladder  M64.4 Maintenance of prosthetic collar around outlet of male bladder  M64.5 Removal of prosthetic collar from around outlet of male bladder  M64.6 Reconstruction of neck of male bladder NEC  M64.7 Introduction of transobturator sling | 0  0  0  0  0  0 | 0  0  0  0  0  0 |
| Open operations on outlet of male bladder | M60.1 Insertion of male retropubic continence device NEC  M60.2 Removal of male retropubic device NEC  M60.3 Removal of artificial urinary sphincter from outlet of male bladder  M60.8 Other specified open operations on outlet of male bladder  M60.9 Unspecified open operations on outlet of male bladder | 0  0  0  0  0 | 0  0  0  0  0 |
| Open excision of prostate | M61.1 Total excision of prostate and capsule of prostate  M61.4 Perineal prostatectomy  M61.8 Other specified open excision of prostate  M61.9 Unspecified open excision of prostate | 2  0  0  1 | 2  0  0  1 |
| Biopsy | M70.2 Perineal needle biopsy of prostate  M70.3 Rectal needle biopsy of prostate | 13  4 | 13  4 |
| Other operations on outlet of male bladder | M70.1 Aspiration of prostate NEC | 0 | 0 |
|  | M70.5 Massage of prostate | 0 | 0 |
|  | M70.6 Radioactive seed implantation into prostate | 0 | 0 |
|  | M70.7 Transurethral radiofrequency needle ablation of prostate | 6 | 6 |
|  | M70.8 Other specified other operations on outlet of male bladder | 8 | 8 |
|  | M70.9 Unspecified other operations on outlet of male bladder | 0 | 0 |
| Other operations on prostate | M71.1 High intensity focused ultrasound of prostate | 0 | 0 |
|  | M71.2 Implantation of radioactive substance into prostate | 0 | 0 |
|  | M71.8 Other specified other operations on prostate | 0 | 0 |
|  | M71.9 Unspecified other operations on prostate | 0 | 0 |
