# Supplementary material for: Prostatic urethral lift (UroLift): a real-world analysis of outcomes using hospital episodes statistics
Source: BMC Urol. 2021 Apr 7;21:55. doi: 10.1186/s12894-021-00824-5 (PMC8028737; doi:10.1186/s12894-021-00824-5)

Online Resource 4: Eligible UroLift procedure uptake in NHS hospitals in England between April 2017 and January 2020.


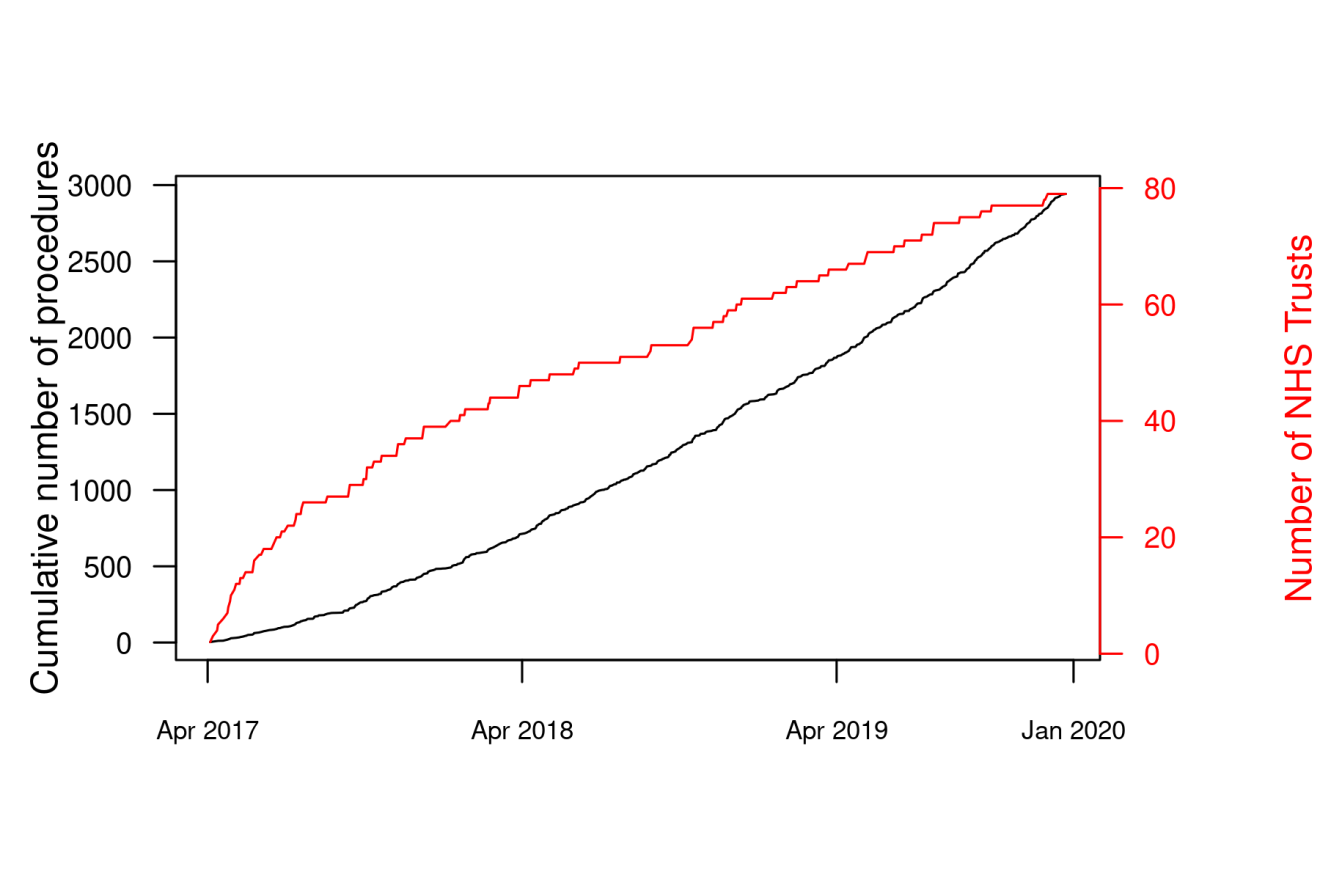

Supplement: Supplementary file 4 — Additional file 4. Online Resource 4: Eligible UroLift procedure uptake in NHS hospitals in England between April 2017 and January 2020. [file 12894_2021_824_MOESM4_ESM.docx]
