# Supplementary material for: Prostatic urethral lift (UroLift): a real-world analysis of outcomes using hospital episodes statistics
Source: BMC Urol. 2021 Apr 7;21:55. doi: 10.1186/s12894-021-00824-5 (PMC8028737; doi:10.1186/s12894-021-00824-5)
Supplement: Supplementary file 5 — Additional file 5. Online Resource 5: In-hospital complications recorded during UroLift implantation. Note complications are not mutually exclusive as patients can have multiple complications. [file 12894_2021_824_MOESM5_ESM.docx]

**Online Resource 5:** In-hospital complications recorded during UroLift implantation. Note complications are not mutually exclusive as patients can have multiple complications.

| Frequency | (%) | ICD10 codes | Description |
| --- | --- | --- | --- |
| 2843 | (96.6%) | None | NA |
| 40 | (1.4%) | R33X | Retention of urine |
| 15 | (0.5%) | R31X | Unspecified haematuria |
| 11 | (0.4%) | T810 | Haemorrhage and haematoma complicating a procedure, not elsewhere classified |
| 8 | (0.3%) | E890 | Postprocedural hypothyroidism |
| 7 | (0.2%) | N328 | Other specified disorders of bladder |
| 2 | (0.1%) | N308 | Other cystitis |
| 2 | (0.1%) | T835 | Infection and inflammatory reaction due to prosthetic device, implant and graft in urinary system |
| 1 | (0.0%) | D649 | Anaemia, unspecified |
| 1 | (0.0%) | E893 | Postprocedural hypopituitarism |
| 1 | (0.0%) | H598 | Other postprocedural disorders of eye and adnexa |
| 1 | (0.0%) | I781 | Naevus, non-neoplastic |
| 1 | (0.0%) | I878 | Other specified disorders of veins |
| 1 | (0.0%) | N390 | Urinary tract infection, site not specified |
| 1 | (0.0%) | N62X | Hypertrophy of breast |
| 1 | (0.0%) | R030 | Elevated blood-pressure reading, without diagnosis of hypertension |
| 1 | (0.0%) | R11X | Nausea and vomiting |
| 1 | (0.0%) | R35X | Polyuria |
| 1 | (0.0%) | R391 | Other difficulties with micturition |
| 1 | (0.0%) | R398 | Other and unspecified symptoms and signs involving the urinary system |
| 1 | (0.0%) | R471 | Dysarthria and anarthria |
| 1 | (0.0%) | S372 | Injury of bladder |
| 1 | (0.0%) | T812 | Accidental puncture and laceration during a procedure, not elsewhere classified |
| 1 | (0.0%) | T815 | Foreign body accidentally left in body cavity or operation wound following a procedure |
| 1 | (0.0%) | T818 | Other complications of procedures, not elsewhere classified |
| 1 | (0.0%) | T828 | Other specified complications of cardiac and vascular prosthetic devices, implants and grafts |
| 1 | (0.0%) | T838 | Other complications of genitourinary prosthetic devices, implants and grafts |
| 1 | (0.0%) | T839 | Unspecified complication of genitourinary prosthetic device, implant and graft |
