# Supplementary material for: Prostatic urethral lift (UroLift): a real-world analysis of outcomes using hospital episodes statistics
Source: BMC Urol. 2021 Apr 7;21:55. doi: 10.1186/s12894-021-00824-5 (PMC8028737; doi:10.1186/s12894-021-00824-5)
Supplement: Supplementary file 6 — Additional file 6. Online Resource 6: Reason for readmissions occurring within 30 days (using first diagnosis code). [file 12894_2021_824_MOESM6_ESM.docx]

Online Resource 6: Reason for readmissions occurring within 30 days (using first diagnosis code).

| \| **ICD10 code** \| **Description** \| **Freq** \| \| --- \| --- \| --- \| \| Z466 \| Fitting and adjustment of urinary device \| 107 \| \| R33X \| Retention of urine \| 65 \| \| R31X \| Unspecified haematuria \| 57 \| \| N40X \| Hyperplasia of prostate \| 27 \| \| N185 \| Chronic kidney disease, stage 5 \| 17 \| \| N328 \| Other specified disorders of bladder \| 17 \| \| N390 \| Urinary tract infection, site not specified \| 16 \| \| T830 \| Mechanical complication of urinary (indwelling) catheter \| 14 \| \| T838 \| Other complications of genitourinary prosthetic devices, implants and grafts \| 7 \| \| T810 \| Haemorrhage and haematoma complicating a procedure, not elsewhere classified \| 6 \| \| R398 \| Other and unspecified symptoms and signs involving the urinary system \| 5 \| \| A419 \| Sepsis, unspecified \| 4 \| \| E834 \| Disorders of magnesium metabolism \| 4 \| \| J181 \| Lobar pneumonia, unspecified \| 4 \| \| I214 \| Acute subendocardial myocardial infarction \| 3 \| \| K409 \| Unilateral or unspecified inguinal hernia, without obstruction or gangrene \| 3 \| \| K635 \| Polyp of colon \| 3 \| \| N179 \| Acute renal failure, unspecified \| 3 \| \| N459 \| Orchitis, epididymitis and epididymo-orchitis without abscess \| 3 \| \| R073 \| Other chest pain \| 3 \| \| R103 \| Pain localized to other parts of lower abdomen \| 3 \| \| R35X \| Polyuria \| 3 \| \| R69X \| Unknown and unspecified causes of morbidity \| 3 \| \| C787 \| Secondary malignant neoplasm of liver and intrahepatic bile duct \| 2 \| \| D649 \| Anaemia, unspecified \| 2 \| \| E831 \| Disorders of iron metabolism \| 2 \| \| E871 \| Hypo-osmolality and hyponatraemia \| 2 \| \| I251 \| Atherosclerotic heart disease \| 2 \| \| I635 \| Cerebral infarction due to unspecified occlusion or stenosis of cerebral arteries \| 2 \| \| K590 \| Constipation \| 2 \| \| K800 \| Calculus of gallbladder with acute cholecystitis \| 2 \| \| M169 \| Coxarthrosis, unspecified \| 2 \| \| M179 \| Gonarthrosis, unspecified \| 2 \| \| M480 \| Spinal stenosis \| 2 \| \| M751 \| Rotator cuff syndrome \| 2 \| \| R060 \| Dyspnoea \| 2 \| \| R074 \| Chest pain, unspecified \| 2 \| \| R300 \| Dysuria \| 2 \| \| Z090 \| Follow-up examination after surgery for other conditions \| 2 \| \| Z436 \| Attention to other artificial openings of urinary tract \| 2 \| \| A099 \| Gastroenteritis and colitis of unspecified origin \| 1 \| \| A411 \| Sepsis due to other specified staphylococcus \| 1 \| \| A415 \| Sepsis due to other Gram-negative organisms \| 1 \| \| A498 \| Other bacterial infections of unspecified site \| 1 \| \| C433 \| Malignant neoplasm: Malignant melanoma of other and unspecified parts of face \| 1 \| \| C443 \| Malignant neoplasm: Skin of other and unspecified parts of face \| 1 \| \| C672 \| Malignant neoplasm: Lateral wall of bladder \| 1 \| \| C674 \| Malignant neoplasm: Posterior wall of bladder \| 1 \| \| C679 \| Malignant neoplasm: Bladder, unspecified \| 1 \| \| C821 \| Follicular lymphoma grade II \| 1 \| \| D128 \| Benign neoplasm: Rectum \| 1 \| \| E001 \| Congenital iodine-deficiency syndrome, myxoedematous type \| 1 \| \| E859 \| Amyloidosis, unspecified \| 1 \| \| E86X \| Volume depletion \| 1 \| \| G618 \| Other inflammatory polyneuropathies \| 1 \| \| G971 \| Other reaction to spinal and lumbar puncture \| 1 \| \| H251 \| Senile nuclear cataract \| 1 \| \| H268 \| Other specified cataract \| 1 \| \| H269 \| Cataract, unspecified \| 1 \| \| H353 \| Degeneration of macula and posterior pole \| 1 \| \| I471 \| Supraventricular tachycardia \| 1 \| \| I472 \| Ventricular tachycardia \| 1 \| \| I500 \| Congestive heart failure \| 1 \| \| I634 \| Cerebral infarction due to embolism of cerebral arteries \| 1 \| \| I859 \| Oesophageal varices without bleeding \| 1 \| \| I861 \| Scrotal varices \| 1 \| \| J22X \| Unspecified acute lower respiratory infection \| 1 \| \| J339 \| Nasal polyp, unspecified \| 1 \| \| J343 \| Hypertrophy of nasal turbinates \| 1 \| \| J380 \| Paralysis of vocal cords and larynx \| 1 \| \| J383 \| Other diseases of vocal cords \| 1 \| \| J440 \| Chronic obstructive pulmonary disease with acute lower respiratory infection \| 1 \| \| J939 \| Pneumothorax, unspecified \| 1 \| \| J969 \| Respiratory failure, unspecified \| 1 \| \| K219 \| Gastro-oesophageal reflux disease without oesophagitis \| 1 \| \| K298 \| Duodenitis \| 1 \| \| K317 \| Polyp of stomach and duodenum \| 1 \| \| K439 \| Other and unspecified ventral hernia without obstruction or gangrene \| 1 \| \| K469 \| Unspecified abdominal hernia without obstruction or gangrene \| 1 \| \| K514 \| Inflammatory polyps \| 1 \| \| K529 \| Noninfective gastroenteritis and colitis, unspecified \| 1 \| \| K573 \| Diverticular disease of large intestine without perforation or abscess \| 1 \| \| K626 \| Ulcer of anus and rectum \| 1 \| \| K640 \| First degree haemorrhoids \| 1 \| \| M119 \| Crystal arthropathy, unspecified \| 1 \| \| M161 \| Other primary coxarthrosis \| 1 \| \| M171 \| Other primary gonarthrosis \| 1 \| \| M199 \| Arthrosis, unspecified \| 1 \| \| M254 \| Effusion of joint \| 1 \| \| M255 \| Pain in joint \| 1 \| \| M258 \| Other specified joint disorders \| 1 \| \| M332 \| Polymyositis \| 1 \| \| M503 \| Other cervical disc degeneration \| 1 \| \| M541 \| Radiculopathy \| 1 \| \| M754 \| Impingement syndrome of shoulder \| 1 \| \| M841 \| Nonunion of fracture [pseudarthrosis] \| 1 \| \| M940 \| Chondrocostal junction syndrome [Tietze] \| 1 \| \| N12X \| Tubulo-interstitial nephritis, not specified as acute or chronic \| 1 \| \| N135 \| Kinking and stricture of ureter without hydronephrosis \| 1 \| \| N200 \| Calculus of kidney \| 1 \| \| N210 \| Calculus in bladder \| 1 \| \| N419 \| Inflammatory disease of prostate, unspecified \| 1 \| \| N428 \| Other specified disorders of prostate \| 1 \| \| N429 \| Disorder of prostate, unspecified \| 1 \| \| N47X \| Redundant prepuce, phimosis and paraphimosis \| 1 \| \| N488 \| Other specified disorders of penis \| 1 \| \| R072 \| Precordial pain \| 1 \| \| R101 \| Pain localized to upper abdomen \| 1 \| \| R104 \| Other and unspecified abdominal pain \| 1 \| \| R13X \| Dysphagia \| 1 \| \| R410 \| Disorientation, unspecified \| 1 \| \| R509 \| Fever, unspecified \| 1 \| \| R55X \| Syncope and collapse \| 1 \| \| S003 \| Superficial injury of nose \| 1 \| \| S008 \| Superficial injury of other parts of head \| 1 \| \| S099 \| Unspecified injury of head \| 1 \| \| S800 \| Contusion of knee \| 1 \| \| T783 \| Angioneurotic oedema \| 1 \| \| T818 \| Other complications of procedures, not elsewhere classified \| 1 \| \| T828 \| Other specified complications of cardiac and vascular prosthetic devices, implants and grafts \| 1 \| \| T848 \| Other complications of internal orthopaedic prosthetic devices, implants and grafts \| 1 \| \| T856 \| Mechanical complication of other specified internal prosthetic devices, implants and grafts \| 1 \| \| T857 \| Infection and inflammatory reaction due to other internal prosthetic devices, implants and grafts \| 1 \| \| Z039 \| Observation for suspected disease or condition, unspecified \| 1 \| \| Z098 \| Follow-up examination after other treatment for other conditions \| 1 \| |
| --- | --- | --- | --- | --- | --- | --- | --- | --- | --- | --- | --- | --- | --- | --- | --- | --- | --- | --- | --- | --- | --- | --- | --- | --- | --- | --- | --- | --- | --- | --- | --- | --- | --- | --- | --- | --- | --- | --- | --- | --- | --- | --- | --- | --- | --- | --- | --- | --- | --- | --- | --- | --- | --- | --- | --- | --- | --- | --- | --- | --- | --- | --- | --- | --- | --- | --- | --- | --- | --- | --- | --- | --- | --- | --- | --- | --- | --- | --- | --- | --- | --- | --- | --- | --- | --- | --- | --- | --- | --- | --- | --- | --- | --- | --- | --- | --- | --- | --- | --- | --- | --- | --- | --- | --- | --- | --- | --- | --- | --- | --- | --- | --- | --- | --- | --- | --- | --- | --- | --- | --- | --- | --- | --- | --- | --- | --- | --- | --- | --- | --- | --- | --- | --- | --- | --- | --- | --- | --- | --- | --- | --- | --- | --- | --- | --- | --- | --- | --- | --- | --- | --- | --- | --- | --- | --- | --- | --- | --- | --- | --- | --- | --- | --- | --- | --- | --- | --- | --- | --- | --- | --- | --- | --- | --- | --- | --- | --- | --- | --- | --- | --- | --- | --- | --- | --- | --- | --- | --- | --- | --- | --- | --- | --- | --- | --- | --- | --- | --- | --- | --- | --- | --- | --- | --- | --- | --- | --- | --- | --- | --- | --- | --- | --- | --- | --- | --- | --- | --- | --- | --- | --- | --- | --- | --- | --- | --- | --- | --- | --- | --- | --- | --- | --- | --- | --- | --- | --- | --- | --- | --- | --- | --- | --- | --- | --- | --- | --- | --- | --- | --- | --- | --- | --- | --- | --- | --- | --- | --- | --- | --- | --- | --- | --- | --- | --- | --- | --- | --- | --- | --- | --- | --- | --- | --- | --- | --- | --- | --- | --- | --- | --- | --- | --- | --- | --- | --- | --- | --- | --- | --- | --- | --- | --- | --- | --- | --- | --- | --- | --- | --- | --- | --- | --- | --- | --- | --- | --- | --- | --- | --- | --- | --- | --- | --- | --- | --- | --- | --- | --- | --- | --- | --- | --- | --- | --- | --- | --- | --- | --- | --- | --- | --- | --- | --- | --- | --- | --- | --- | --- | --- | --- | --- | --- | --- | --- | --- | --- | --- | --- | --- | --- | --- | --- | --- | --- | --- | --- | --- | --- | --- | --- | --- | --- | --- | --- | --- | --- | --- | --- | --- | --- | --- | --- | --- | --- | --- | --- | --- |
