# Supplementary material for: Prostatic urethral lift (UroLift): a real-world analysis of outcomes using hospital episodes statistics
Source: BMC Urol. 2021 Apr 7;21:55. doi: 10.1186/s12894-021-00824-5 (PMC8028737; doi:10.1186/s12894-021-00824-5)
Supplement: Supplementary file 7 — Additional file 7. Online Resource 7: The main procedure conducted during urology outpatient appointment conducted within 30 days (using first procedure code). [file 12894_2021_824_MOESM7_ESM.docx]

Online Resource 7: The main procedure conducted during urology outpatient appointment conducted within 30 days (using first procedure code).

| OPCS | Description | Freq |
| --- | --- | --- |
| M473 | Removal of urethral catheter from bladder | 106 |
| U124 | Ultrasound of bladder | 32 |
| X621 | Assessment by uniprofessional team NEC | 15 |
| U262 | Uroflowmetry NEC | 11 |
| M479 | Unspecified urethral catheterisation of bladder | 9 |
| M459 | Unspecified diagnostic endoscopic examination of bladder | 5 |
| U216 | Ultrasound scan NEC | 3 |
| X623 | Assessment by multidisciplinary team NEC | 3 |
| M472 | Change of urethral catheter into bladder | 2 |
| U082 | Ultrasound of abdomen | 2 |
| U263 | Test strip urinalysis | 2 |
| M471 | Urethral irrigation of bladder | 1 |
| M474 | Urodynamic studies using catheter | 1 |
| M494 | Introduction of therapeutic substance into bladder | 1 |
| U093 | Magnetic resonance imaging of pelvis | 1 |
| U101 | Cardiac computed tomography for calcium scoring | 1 |
| U128 | Other specified diagnostic imaging of genitourinary system | 1 |
| U199 | Unspecified diagnostic electrocardiography | 1 |
| U264 | Urodynamics NEC | 1 |
| U332 | Application of ambulatory blood pressure monitor | 1 |
