# Supplementary material for: Prostatic urethral lift (UroLift): a real-world analysis of outcomes using hospital episodes statistics
Source: BMC Urol. 2021 Apr 7;21:55. doi: 10.1186/s12894-021-00824-5 (PMC8028737; doi:10.1186/s12894-021-00824-5)
Supplement: Supplementary file 8 — Additional file 8. Online Resource 8: Retreatment procedures captured during follow-up. [file 12894_2021_824_MOESM8_ESM.docx]

Online Resource 8: Retreatment procedures captured during follow-up

| Procedure | Patients |
| --- | --- |
| Secondary UroLift procedure | 57 (4.2%) |
| - removal | 0 |
| - renewal | 3 |
| - adjustment | 12 |
| - further implantation | 43 |
| Endoscopic intervention | 158 (5.4%) |
| - Endoscopic resection outlet of male bladder | 134 |
| - Other therapeutic endoscopic operations on outlet of male bladder | 37 |
| Open intervention | 0 (0%) |
