# Supplementary material for: Prostatic urethral lift (UroLift): a real-world analysis of outcomes using hospital episodes statistics
Source: BMC Urol. 2021 Apr 7;21:55. doi: 10.1186/s12894-021-00824-5 (PMC8028737; doi:10.1186/s12894-021-00824-5)

Online Resource 8: Hazard rate of retreatment (subsequent UroLift intervention or other endoscopic prostate intervention) following index UroLift implantation procedure. [Note: due to limited number of events kernel-density estimated smoothing was not appropriate, and piecewise exponential hazard function was applied]


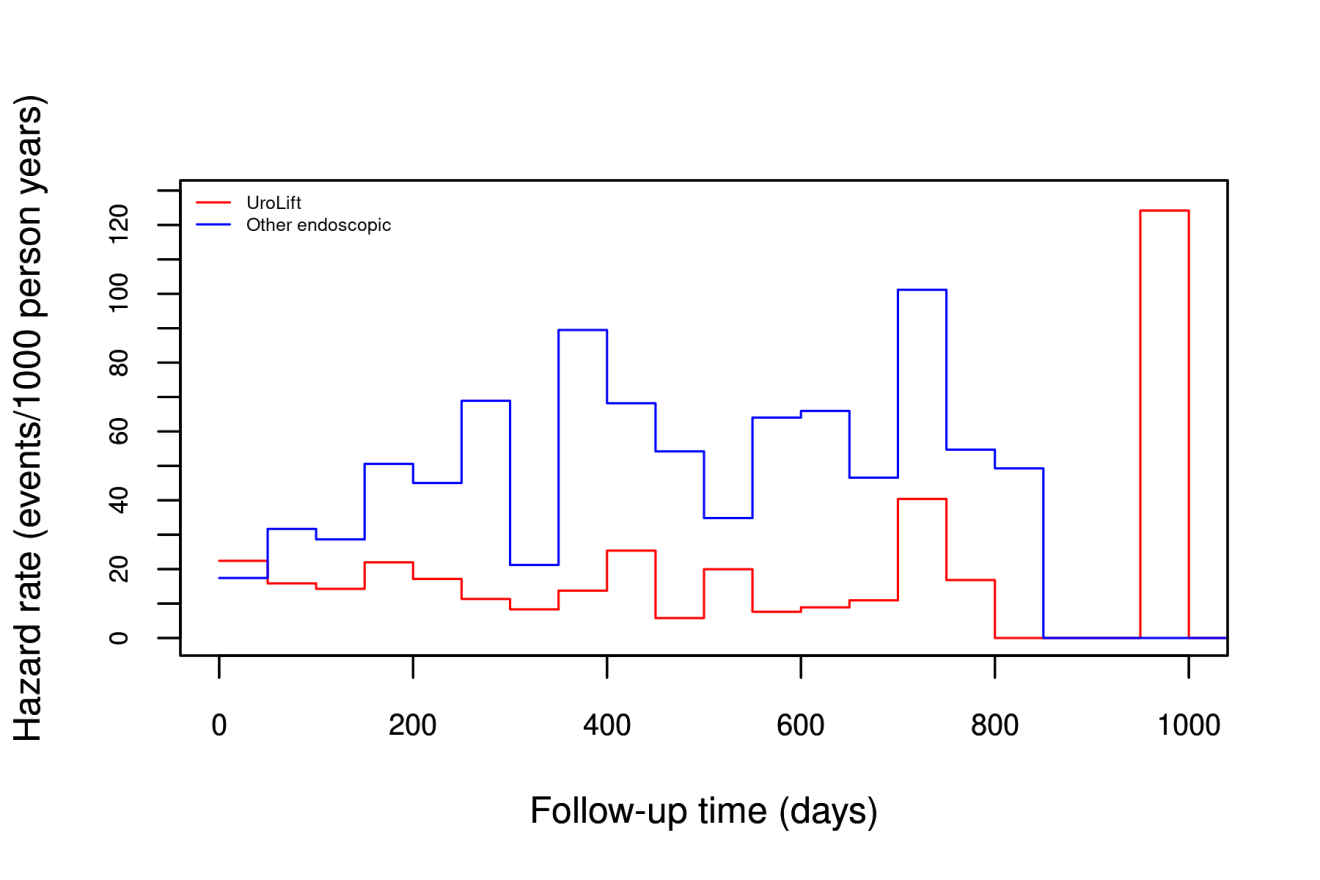

Supplement: Supplementary file 9 — Additional file 9. Online Resource 9: Hazard rate of retreatment (subsequent UroLift intervention or other endoscopic prostate intervention) following index UroLift implantation procedure. [Note: due to limited number of events kernel-density estimated smoothing was not appropriate, and piecewise exponential hazard function was applied]. [file 12894_2021_824_MOESM9_ESM.docx]
